# Factors associated with recurrence in operable breast cancer patients undergoing surgery as the sole treatment modality

**DOI:** 10.1590/acb402025

**Published:** 2025-02-24

**Authors:** Maressa Daniela Anghinoni Bonissoni, Fernanda Mara Alves, Rafaella Frederico Almeida, Lucca Smaniotto, Maria Paula de Andrade Berny, Victor Pereira da Silva, Brenda Stephanie Fiuza, Guilherme Cirino Rodrigues, Eloisa Maria Neres, Rodrigo Kern, Carlos Victor Pereira dos Santos, Janoário Athanazio de Souza, Daniel Rech, Carolina Panis

**Affiliations:** 1 Universidade Estadual do Oeste do Paraná – Centro de Ciências da Saúde – Programa de Residência Médica em Cirurgia Geral – Francisco Beltrão (PR) – Brazil.; 2 Universidade Estadual do Oeste do Paraná – Centro de Ciências da Saúde – Laboratório de Biologia de Tumores – Francisco Beltrão (PR) – Brazil.; 3 Hospital de Câncer de Francisco Beltrão – Francisco Beltrão (PR) – Brazil.

**Keywords:** Breast Neoplasms, Therapeutics, General Surgery, Recurrence

## Abstract

**Purpose:**

Approximately 10% of breast cancer patients with localized operable disease experience recurrence. This study aimed to evaluate patients with early-stage breast cancer (TNM I and II) who underwent surgery without the need for neoadjuvant therapy.

**Methods:**

The total of 117 patients was included in the study: 104 without recurrence and 13 with recurrence. We analyzed various clinicopathological parameters, including body mass index, age at diagnosis, menopausal status, pesticide exposure, and tumor characteristics such as HER2 amplification, Ki67 index, molecular subtype, and histological grade.

**Results:**

Two primary subsets were identified as being associated with disease recurrence. The first subset (PC1) was characterized by HER2 amplification and metastatic disease, while the second subset (PC2) comprised premenopausal young women exposed to pesticides who had triple-negative tumors. While some of these factors are well-documented in the literature, pesticide exposure emerged as a notable regional factor contributing to poor outcomes in breast cancer patients.

**Conclusion:**

These findings underscored the significance of identifying local and regional risk factors when assessing recurrence risk in women with breast cancer, particularly in cases in which surgery is the primary treatment approach.

## Introduction

Breast cancer remains one of the major global health concerns, affecting millions of women worldwide annually. Significant advancements have been made in early diagnosis and treatment of early-stage breast cancer, leading to higher survival rates and improved quality of life for patients^1^. However, we still face the challenge of tumor recurrence, which can occur even after apparently successful treatment^2^. Understanding the factors associated with this recurrence, especially in patients with early-stage tumors, is essential for developing strategies to predict and rapidly identify recurrences, as well as modify intervenable risk factors.

In assessing breast cancer patients, certain factors may indicate a higher risk of recurrence. These factors are divided into those related to the patient, such as young age at diagnosis, premenopausal status, and obesity. Although some of these factors, like age and menopausal status, cannot be modified, obesity can be a focus of attention during treatment^3^. Other predictive factors for poor outcomes are related to tumor analysis, such as HER2 amplification, high Ki67 index, and the presence of angiolymphatic emboli. Subtype-related factors are also unmodifiable. Nevertheless, even these immutable factors provide important data for better patient monitoring^4^.

When considering treatment for patients with early-stage breast tumors without metastases and low histological grades, the standard approach is surgery without the need for neoadjuvant therapy^5^. Depending on the surgical modality adopted and definitive biopsy results, adjuvant treatment may be recommended for these patients^6^. Patients with more advanced tumors require neoadjuvant therapy. Even patients with early-stage tumors undergoing the gold-standard treatment–surgery, with or without adjuvant therapy–have a risk of recurrence^2^^,^ 7.

In this context, this study explored the variables associated with recurrence in patients with early-stage breast tumors and the possible clinicopathological parameters related to this outcome.

## Methods

This proposal has been approved by the Institutional Ethics Committee under protocol number Certificate of Presentation of Ethical Appreciation 35524814.4.0000.0107, with opinion number 810.501. Only patients who signed the informed consent form were included.

This study is a retrospective analysis with primary data collection from women treated at the Cancer Hospital of Francisco Beltrão, PR, Brazil, between 2015 and 2022, totaling 800 patients. Inclusion criteria were patients who underwent surgery without neoadjuvant therapy and had follow-up until the data collection date. Exclusion criteria included patients who underwent neoadjuvant therapy, loss to follow-up during the period, or death from causes other than tumor recurrence or disease progression. Additionally, patients whose biopsy did not indicate breast cancer were excluded.

For recurrence characterization, patients with evidence of new tumor lesions in breasts or other sites with biopsy compatible with breast cancer recurrence were considered. Thus, after exclusions, a total of 117 women was included in this study, categorized into groups with recurrence (n = 13) and without recurrence (n = 104).

We collected the following information from medical records: expression of hormonal receptors (immunohistochemical analysis for estrogen – ER, and progesterone – PR receptors, categorized as presence or absence), human epidermal growth receptor 2 (HER2) amplification (evaluated by *in-situ* hybridization, categorized as presence or absence), histological grade of tumors (categorized as low for grades 1 and 2, and high for grade 3 tumors), presence of metastases in axillary lymph nodes (categorized as presence or absence), tumor size (categorized as less or equal/higher than 20 mm), molecular subtype of breast cancer (categorized as luminal A – ER and/or PR positive, ki 67 index under 14%, HER2 negative; luminal B - A – ER and/or PR positive, ki 67 index ≥ 14%, HER2 negative; HER2-amplified - A – ER and/or PR negative, any ki 67 index value HER2 positive; and triple-negative - A – ER and/or PR negative, any ki 67 index value, HER2 negative), age at diagnosis, body mass index (BMI), menopausal status at diagnosis (categorized as presence or absence), risk stratification for death and recurrence (categorized as low, intermediate or high risk), disease recurrence in five years, distant sites of metastasis, and survival profile in five years^8^.

To obtain the occupational exposure profile to pesticides, patients were interviewed. A validated instrument for this purpose was subsequently used to characterize occupational exposure to pesticides. Exposure criteria were based on continuous, unprotected, and direct handling of pesticides. Rural women with a history of direct handling of pesticides without using protective gloves during preparation and dilution of the poison solution, pesticide application, and/or decontamination of personal protective equipment, and/or washing of clothes used during spraying, who reported living at least 50% of their lives under direct pesticide handling at least twice a week throughout the year were considered exposed. Urban workers with no previous or current history of occupational exposure to pesticides were considered non exposed^9^^,^ 10.

**Figure 1:**
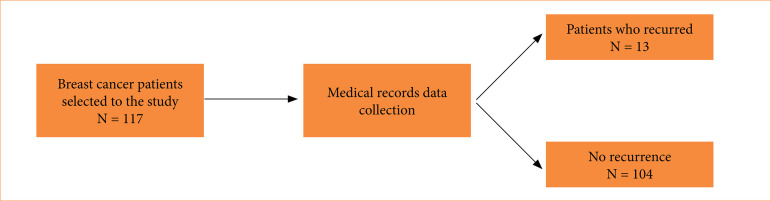
Design of the study.

Source: Elaborated by the authors.

The study design is illustrated in Fig. 1.

The statistical study aimed to assess differences between breast cancer patients who underwent surgery and experienced recurrence and those without a history of recurrence. From the data obtained and properly tabulated in Microsoft Excel spreadsheets, χ^2^ tests of independence were conducted to assess the association between different descriptive variables of the samples and cancer-related variables and their association with recurrence or non-recurrence. When necessary, this was followed by the adjusted residual post-test, which allows for the identification of which categories the variables present statistical association. The statistical analyses were performed using the XLSTAT statistical software (ADDINSOFT, 2018), assuming a significance level of 5% for all analyses.

Subsequently, the variables were analyzed through principal component analysis (PCA). In PCA, factorial loads are established, which are defined as the correlations of each variable with the factor composition, with the factor being a new statistical variable defined by the set of factorial loads. Spearman correlation analyses were also conducted between the evaluated parameters using the GraphPad Prism software version 9.0 (Graphpad Software, San Diego, CA, United States of America).

## Results

When evaluating the profile of the patients included in the study, it was identified that 29% of them were under 50 years old at diagnosis, 56.4% had a positive family history of neoplasia, and 30% were not in menopause. Additionally, 57.2% of the patients were obese. Exposure to pesticides, a prevalent factor in the region, affected 64.1% of the patients. In an initial risk stratification assessment, 21.3% of the patients were considered at high risk for recurrence.

Analyzing the tumor profile of this population, 5.9% of tumors showed HER2 amplification, and 58.9% had high Ki67, while 57.2% of tumors belonged to molecular subtypes different from luminal A. Moreover, 24.7% of tumors presented angiolymphatic emboli. From the data evaluation, a recurrence rate of 11.1% was identified in the service.

When associating the descriptive variables of the samples, it was noticed that none of them showed a statistically significant association with recurrence (*p* > 0.05, Tables 1 and 2). In other words, no significant differences were found between the groups with and without recurrence.

**Table 1:** Absolute frequencies and relative frequencies of descriptive qualitative variables. χ^2^ test of independence.

Variable	Category	No			Yes		*p*-value
		N	%		N	%	
**Age at diagnosis**	> 50 years old	73	70.87		9	69.23	0.902
	≤ 50 years old	30	29.13		4	30.77	
**Family history**	No	40	41.24		4	30.77	0.469
	Yes	57	58.76		9	69.23	
**Menopause**	No	29	28.71		3	23.08	0.670
	Yes	72	71.29		10	76.92	
**Body mass index**	Overweight	58	71.60		9	75.00	0.807
	Normal	23	28.40		3	25.00	
**Pesticide exposure**	No	35	35.35		2	15.38	0.150
	Yes	64	64.65		11	84.62	

Source: Elaborated by the authors.

**Table 2:** Absolute frequencies and relative frequencies of qualitative variables related to cancer. χ^2^ test of independence.

Variable	Category	No			Yes		*p*-value
		N	%		N	%	
**Estrogen receptors**	Negative	21	20.39		4	30.77	0.391
	Positive	82	79.61		9	69.23	
**Progesterone receptors**	Negative	38	37.62		7	53.85	0.260
	Positive	63	62.38		6	46.15	
**HER-2 amplification**	No	96	95.05		11	84.62	0.140
	Yes	5	4.95		2	15.38	
**KI67 index (%)**	< 14	42	41.58		2	16.67	0.094
	≥ 14	59	58.42		10	83.33	
**Molecular subtype**	Luminal A	44	43.56		3	23.08	0.158
	Others	57	56.44		10	76.92	
**Tumor size (mm)**	> 20	66	66.00		10	76.92	0.430
	≤ 20	34	34.00		3	23.08	
**Tumor grade**	1 and 2	85	83.33		8	61.54	0.059
	3	17	16.67		5	38.46	
**Intratumoral emboli**	Absence	70	73.68		8	66.67	0.606
	Presence	25	26.32		4	33.33	
**Lymphnodal invasion**	No	7	8.24		1	9.09	0.923
	Yes	78	91.76		10	90.91	
**Distant metastasis**	No	50	62.50		5	45.45	0.278
	Yes	30	37.50		6	54.55	
**Risk stratification for reccurrence and death**	High risk	21	22.11		4	33.33	0.386
	Low risk	74	77.89		8	66.67	

Source: Elaborated by the authors.

A Spearman correlation analysis was conducted between the data of patients with recurrence to verify significant correlations among the evaluated parameters, considering individual patient characteristics. As observed in Table 3, there were significant positive correlations between the Ki67 proliferation index and the presence of HER2 amplification, between the Ki67 proliferation index and molecular subtype, between the molecular subtype and BMI (overweight), and between risk stratification and BMI (overweight). These correlations indicate that, in these specific cases, certain tumor parameters and individual patient characteristics are significantly associated, providing important insights into the factors influencing breast cancer recurrence.

**Table 3:** Significant Spearman’s correlations among variables in the patient group with recurrence.

Correlation	R	95% Connfidence interval	*p*-value
Ki67 index vs. HER2-amplification	0.6547	0.1103–0.8969	0.0303
Ki67 index vs. molecular subtype	0.6653	0.1289–0.9005	0.0202
Emboli vs. molecular subtype	0.7009	0.1940–0.9124	0.0222
Molecular subtype vs. body mass index	0.7274	0.2454–0.9210	0.0096
Risk stratification vs. weight	0.5964	0.01487–0.8764	0.0471

Source: Elaborated by the authors.

**Figure 2:**
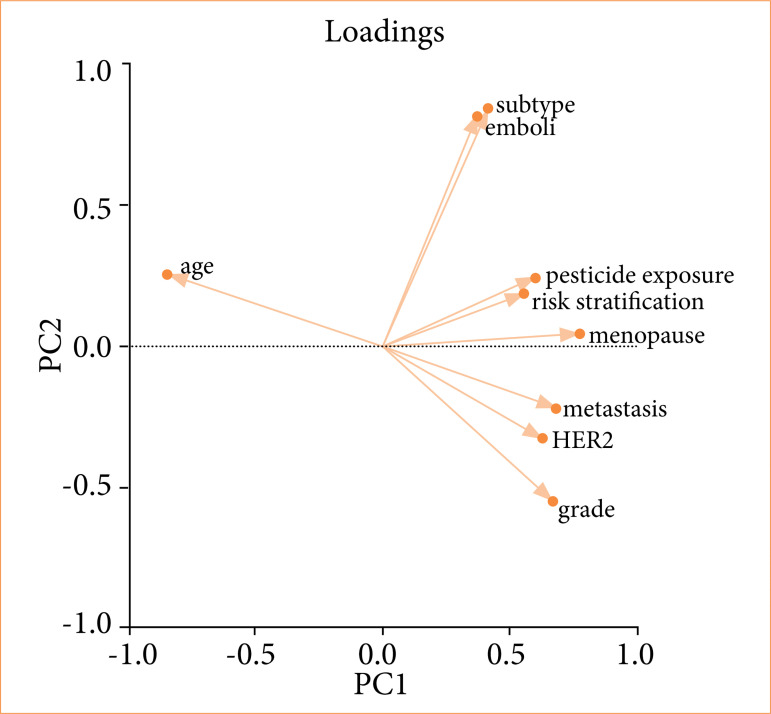
Principal component analysis in patients who recurred after breast cancer surgery.

Source: Elaborated by the authors.

In the principal component (PC) analysis (Fig. 2), we aimed to understand which factors were influencing breast cancer recurrence. The analysis was conducted only in the group of patients who recurred disease. These clusters emphasize that diverse factors influence breast cancer prognosis in our study, reflecting the disease’s heterogeneity of the studied groups into two distinct clusters (PC1 and PC2).

The first component (PC1) is characterized by grade 3 tumors, HER2 amplification, and distant metastases. It means that, in this group, patients exhibited undifferentiated HER2-amplified tumors that resulted in metastatic disease. This component indicates which parameters are linked to metastasis.

The second component (PC2) included pesticide exposure, triple-negative molecular subtype, angiolymphatic emboli, younger age at diagnosis, and premenopausal status. It indicates that, in this group, premenopausal younger women exposed to pesticides had triple-negative tumors with emboli. This component suggest which factors are linked to early breast cancer.

## Discussion

This study investigated the importance of risk factors in the recurrence rate of surgically treated early-stage breast tumors, aiming to identify causes for this recurrence despite the high-cure rates observed in these cases. It was found that traditional factors such as BMI, age at diagnosis, and menopausal status remain important in predicting recurrence. Additionally, exposure to pesticides emerged as a significant factor in our population study who recurred disease, linked to triple-negative breast tumors in premenopausal young women. This finding is relevant as it suggests the inclusion of this factor in risk stratification and therapeutic decision-making for this patient group.

The cure rate for surgically treated early-stage breast tumors is high, as evidenced by Colleoni et al.^2^, who reported a recurrence rate of 10.4% in a study of 4,000 surgically treated patients, with most recurrences occurring in the first five years of follow-up. The aim of this study was to investigate patients who did not fit this profile and understood the reasons behind their tumor recurrence, as well as to identify non-conventional or regional factors that should be considered in the surgically eligible patient group. In our population, the recurrence rate was 11.1%, very close to the rates documented in the literature.

A meta-analysis of 88 trials involving 62,923 women with ER-positive breast cancer revealed a strong correlation between the risk of disease recurrence and the original tumor-node (TN) status. This finding highlights that recurrence risk is influenced more by the initial TN status than by subsequent treatments, underscoring the critical role of surgery as a treatment modality for breast cancer^11^.

Risk factors related to patients (such as age, family history, menopause, BMI, and pesticide exposure) and tumor characteristics (such as hormone receptor and HER2 expression, Ki67, among others) were evaluated. Due to sample size and similarity of profiles between groups with and without recurrence, statistically significant differences were not identified in this initial analysis, although it is known that age and menopause are risk factors for an unfavorable prognosis. Previous studies, such as those by Bastiaannet et al.^7^, Billena et al.^12^, and Eaker et al.^13^, also observed a higher recurrence rate in younger patients. Additionally, factors such as high Ki67, triple-negative subtype, and HER2 amplification are associated with a higher risk of recurrence.

A study conducted in a Brazilian cohort showed the efficiency of breast-conserving surgery combined to other clinicopathological characteristics with improved survival outcomes in locally advanced breast cancer^14^. Another study in a Brazilian cohort demonstrated that young women diagnosed with breast cancer have aggressive disease at diagnosis, resulting in high rates of death and disease progression^15^. It reinforced the need for identifying further risk factors for understanding breast cancer behavior in young women.

In this context, correlation and PCA allowed for a deeper understanding of the data in breast cancer patients that underwent surgery and recurred, revealing that patients with a combination of risk factors have an increased probability of disease recurrence. For example, as shown in Table 3, patients with high Ki67 and recurrence are more likely to have HER2 amplification and triple-negative molecular subtype. Moreover, patients with triple-negative tumors and recurrence tend to have a higher BMI. The association between molecular subtype and presence of angiolymphatic emboli was also observed. Each of these factors individually contributes to a higher risk of recurrence, but, when combined, they further increase this possibility.

Tumors can recur after surgery due to several events, such as microscopic cancer cells left behind at the surgical site, undetectable micrometastases spreading to distant parts of the body before surgery, or the biological aggressiveness of the cancer. This reinforces why it is important to identify putative risk factors associated to specific population profiles in breast cancer^16^. Additionally, changes in the tissue environment after surgery, such as inflammation, can promote cancer regrowth^17^. Therefore, identifying risk factors associated to recurrence allows its early detection, as well as a comprehensive follow-up care for managing this risk.

Pesticide exposure emerged as a significant risk factor in this population. Many patients evaluated in this study work in environments where they are chronically exposed to these harmful substances. Previous studies have shown that pesticide exposure can alter the molecular behavior of the disease. Additionally, the immunotoxicity caused by pesticide exposure may have important implications for understanding disease progression and tailoring treatment for different patient subgroups in our region^8^^,^ 18.

Although this study identifies associations between risk factors and recurrence rates, it has several limitations. These include a small sample size and the similarity of risk factors observed in both populations–with and without recurrence. Additionally, follow-up is still ongoing, and data on disease-free survival and overall survival remain incomplete. Furthermore, the pesticide exposure data relies on self-reported information rather than direct measurements from blood or urine samples, which did not allow to understand which pesticides are present in this context.

Despite these limitations, the study successfully identified pesticide exposure as a significant risk factor for recurrence, underscoring the need for preventive measures and early screening in this population. While the recurrence rate observed aligns closely with rates reported in the literature, the identification of pesticide exposure as a key risk factor provides a new perspective on breast cancer recurrence within our population.

The clinical relevance of our findings lies in addressing the limited understanding of why some patients, whose tumor characteristics qualify them for surgery alone, do not achieve the expected outcomes. Our study demonstrated that in this group–women with breast cancer treated solely with surgery–there are two distinct clinicopathological profiles. Factors like pesticide exposure appear to influence recurrence in young, premenopausal women, shedding light on disease aggressiveness in specific locoregional contexts. Studies in the Brazilian population have reinforced the importance to focus on early breast cancer detection in younger women19, because this disease is frequently aggressive in young women.

These insights could improve understanding and inform tailored approaches to treatment and follow-up.

## Conclusion

The study underscores the critical importance of thoroughly evaluating clinicopathological characteristics and locoregional factors when determining the recurrence risk in patients with operable breast cancer. By integrating these parameters into risk assessment strategies, clinicians can make more informed decisions regarding treatment planning and follow-up care.

## Data Availability

The data will be available upon request.
